# Enhancing membrane performance for oily wastewater treatment: comparison of PVDF composite membranes prepared by coating, blending, and grafting methods using TiO_2_, BiVO_4_, CNT, and PVP

**DOI:** 10.1007/s11356-024-35456-3

**Published:** 2024-11-14

**Authors:** Erika Nascimben Santos, Ákos Ferenc Fazekas, Laura Fekete, Tímea Miklós, Tamás Gyulavári, Sivasundari Arumugam Gokulakrishnan, Gangasalam Arthanareeswaran, Cecilia Hodúr, Zsuzsanna László, Gábor Veréb

**Affiliations:** 1 Institute of Biosystem Engineering, Faculty of Engineering, University of Szeged, Moszkvai Blvd. 9, HU-6725 Szeged, Hungary; 2 Doctoral School of Environmental Sciences, Faculty of Science and Informatics, University of Szeged, Aradi Vértanúk Sqr. 1, HU-6720 Szeged, Hungary; 3 Department of Applied and Environmental Chemistry, Institute of Chemistry, University of Szeged, Rerrich Béla Sqr. 1, HU-6720 Szeged, Hungary; 4 Department of Chemical Engineering, National Institute of Technology, Membrane Research Laboratory, Tiruchirappalli, 620015 Tamilnadu India

**Keywords:** Membrane filtration, Membrane modification, Nanomaterials, Oil-in-water emulsion, Phase inversion, Photocatalytic activity

## Abstract

This comparative study investigates the modification of polyvinylidene fluoride (PVDF) membranes with different nanoparticles (TiO_2_ or TiO_2_-based composites containing BiVO_4_ and/or CNT), using three distinct methods (blending, coating, and grafting) and polyvinylpyrrolidone (PVP). The objective was to enhance the photocatalytic and filtration performance for the separation of oil-in-water emulsions. Regarding the UV activity, the PVDF-TiO_2_/CNT/PVP-coated membrane presented the best performance. Overall, the addition of 2 wt.% CNT to the TiO_2_ notably enhanced the photocatalytic activity of the membranes for both UV and visible irradiations. Meanwhile, the presence of 2 wt.% BiVO_4_ was beneficial only for photocatalysis under visible light irradiation. Regarding the filtration of the oil-in-water emulsions, 2 wt.% CNT or BiVO_4_ addition resulted in the highest fluxes in the series of the PVDF-TiO_2_-grafted membranes. The presence of pore former PVP led to relatively high fluxes and photocatalytic activities for all series. Regarding the modification methods, coated membranes showed the highest photocatalytic efficiency and lowest fluxes. Grafted membranes showed relatively high photocatalytic efficiencies and the best filtration performances.

## Introduction

Oily wastewater pollution is a pressing environmental concern that demands effective treatment strategies to safeguard water resources. Conventional methods, including physical separation, chemical coagulation, and biological processes, have limitations in the elimination of oily contaminants and often face challenges related to membrane fouling. Consequently, novel approaches integrating advanced materials have garnered substantial attention in recent years (Liang et al. 2022; Nielsen et al. 2023; Nouzaki et al. 2023; Padaki et al. 2015; Yalcinkaya et al. 2020).

Membrane-based processes have emerged as promising techniques for oily wastewater treatment due to their high separation efficiency, low energy consumption, and ease of operation (Syarifah et al. 2017; da Motta et al. 2013; Nascimbén Santos et al. 2020; Munirasu et al. 2016; Ong et al. 2013). Polyvinylidene fluoride (PVDF)—a widely used polymer in membrane fabrication—exhibits excellent chemical resistance, thermal stability, and mechanical strength, but the inherent hydrophobicity and narrow pore size distribution of PVDF membranes limit their application in oily wastewater treatment, primarily due to fouling issues (Safari et al. 2019; Samsudin et al. 2018; Back et al. 2019). To overcome this limitation, researchers have explored various modification strategies.

The utilization of photocatalytic nanoparticles in membrane technology is a promising way to enhance the performance of membrane-based oily wastewater treatment. Among the various photocatalytic nanoparticles, titanium dioxide (TiO_2_) and bismuth vanadate (BiVO_4_) have demonstrated remarkable photocatalytic activity and compatibility with membrane systems. These nanoparticles possess unique physicochemical properties that enable the efficient degradation of oil contaminants under illumination, offering a promising approach to mitigate fouling problems and improve treatment efficiency (Hemavibool et al. 2022; Mesa et al. 2020; Tang et al. 2023; Arast et al. 2023; Zhang et al. 2021).

TiO_2_ is a widely studied photocatalyst known for its excellent chemical stability, strong oxidative properties, and photoactive behavior under UV light irradiation (Leong et al. 2014; Zangeneh et al. 2018; Deng et al. 2021; Venkatesh et al. 2016, 2020; Benhabiles et al. 2019). BiVO_4_ possesses a narrower bandgap, allowing it to efficiently absorb visible light, which comprises a significant portion of solar radiation. This property may enable BiVO_4_ to harness solar energy more effectively for oil degradation (Wetchakun et al. 2015; Zalfani et al. 2015; Song et al. 2017; Arast et al. 2023). Carbon nanotubes (CNTs), with their unique nanostructure and excellent electron transport properties, facilitate efficient charge carrier separation and suppress the recombination of electrons and holes; therefore, they can be used in composites to enhance photocatalytic activity (Sisay et al. 2023; Veréb et al. 2019; Fekete et al. 2023; Liu et al. 2017; Venkatesh et al. 2020). When combined in various proportions, TiO_2_, BiVO_4_, and CNTs can synergistically enhance the performance of PVDF membranes used for the treatment of oily wastewater. The incorporation of these nanomaterials provides additional functionalities such as photocatalytic degradation of organic contaminants, increased hydrophilicity, improved antifouling properties, and enhanced mechanical strength (Sisay et al. 2022, 2023; Arast et al. 2023). Additionally, their unique properties allow for the generation of reactive oxygen species under illumination, which promotes the degradation of organic pollutants. Our research group (Nascimben Santos et al. 2020) has investigated the use of TiO_2_- and BiVO_4_-coated membranes for the treatment of oily wastewater and found that the nanocomposite constituents enhanced the membrane performance. Fekete et al. (Fekete et al. 2023) successfully enhanced the flux of oil-in-water emulsion filtration when using TiO_2_/CNT_2%_-coated membranes and the recovery of the membrane after photocatalytic regeneration.

The different methods for incorporating photocatalytic nanoparticles into membranes (i.e., blending, coating, and grafting) also reveal the distinct advantages and limitations of each approach (Kalantari et al. 2014; Nascimbén Santos et al. 2020; Gokulakrishnan et al. 2021). Blending involves the homogeneous dispersion of nanoparticles within the membrane matrix, resulting in improved photocatalytic activity and oil degradation efficiency. It is a simple and cost-effective approach with good compatibility between the polymer matrix and nanoparticles (Mahdavi et al. 2020; Mansourizadeh and Azad 2014; Ali et al. 2014; Nascimben Santos et al. 2022). Coating, on the other hand, entails the deposition of a thin layer of nanoparticles onto the membrane surface, enhancing oil rejection rates and membrane performance (Lenac et al. 2022; Chen et al. 2021; Kovács et al. 2018; Tang et al. 2023); however, the durability of these coated membranes is limited. Grafting involves anchoring nanoparticles onto the membrane surface, offering strong adhesion and stability, preventing nanoparticle leaching during operation, and providing enhanced photocatalytic performance and improved fouling resistance. Its disadvantage is that it introduces additional steps and chemical reagents into the membrane preparation process (Yalcinkaya et al. 2020; Sun et al. 2018; Yang et al. 2014; Zhao et al. 2014; Madaeni et al. 2011).

Despite the progress made in the field of membrane filtration, several knowledge gaps and challenges remain to be addressed. Further investigation is needed to determine the optimal proportions of TiO_2_, BiVO_4_, and CNT constituents in the membrane matrix to achieve the desired photocatalytic performance (Sisay et al. 2023, 2022). Comparative studies have demonstrated varying effects of nanoparticle ratios on membrane performance, emphasizing the need for a systematic evaluation (Liu et al. 2011; Matos et al. 2016; Nascimben Santos et al. 2022; Ahmad et al. 2016).

In addition to the incorporation of photocatalytic nanoparticles, the use of polyvinylpyrrolidone (PVP) as a membrane modifier has also gained attention due to its ability to enhance membrane performance. PVP, a water-soluble polymer, can improve membrane hydrophilicity, antifouling properties, and oil–water separation efficiency (Ghelich et al. 2019; Nascimben Santos et al. 2022; Tofighy et al. 2021; Mahdavi et al. 2020; Back et al. 2019; Zhou and Xi 2007; Yoo et al. 2004).

All the mentioned nanomaterials have already proved to be beneficial in different conditions (different immobilization methods, irradiations, etc.). Hence, this study aims to compare the advantages and the limitations of different immobilization methods (blending, coating, and grafting) of TiO_2_, TiO_2_/CNT, TiO_2_/BiVO_4_, and TiO_2_/BiVO_4_/CNT nanoparticles in/on PVDF membranes. The addition of PVP was also evaluated in the case of PVDF membranes blended, coated, or grafted with TiO_2_/CNT. Photocatalytic activities were compared under different irradiations (UV, visible, and simulated solar), and the membrane filtration of an oil-in-water emulsion was also investigated in detail.

## Material and methods

### Fabrication of neat membranes

Neat membranes were fabricated using the phase inversion with immersion precipitation method. First, 6.0 g (15 wt.%) of PVDF (Alfa Aesar™, 64.03 kDa, Kandel, Germany) was dried in an oven at 80 °C for 4 h in a glass vessel. Then, 40 g of dope solution was fabricated by the addition of 33.1 mL (85 wt.%) of N-methyl-2-pyrrolidone (NMP; Molar Chemicals Kft., 1.028 g mL^–1^ density, Halásztelek, Hungary) solvent into the dried PVDF-containing glass vessel. The solutions were stirred for 20 h at 20 rpm and 60 °C, followed by 30 min in an ultrasonication bath (Shanghai ZX Trading Co., LT PS-60A, Shanghai, China) at maximal amplitude. After that, the solution was aged for 24 h in the dark at 60 °C to ensure the complete removal of bubbles formed during the previous 20-h-long stirring and ultrasonication.

The dope solutions were set aside until room temperature was reached, and then they were cast on a glass plate at 200 μm thickness using a casting knife (BGD205 type, 160 mm wide, Biuged Laboratory Instruments Ltd., Guangzhou, China). The partial solvent/non-solvent exchange was done by setting aside the glass plate for 30 s for evaporation. Then, the glass plate was immersed into a non-solvent bath containing ultrapure water (PureLab chorus, ELGA, Veolia, Celle, Germany) and 3 g L^–1^ sodium lauryl sulfate surfactant (Molar Chemicals Kft., Halásztelek, Hungary) at 20 °C for 60 min. The surfactant was used to reduce the surface tension at the interface of the polymer and the non-solvent bath, ensuring the formation of a more porous structure, as observed in previous studies (Safari et al. 2019; Arthanareeswaran et al. 2010; Srivastava et al. 2011; Nascimben Santos et al. 2022). After that, the membrane was washed with ultrapure water and kept for 48 h in an ultrapure water bath at room temperature and then dried at room temperature prior to the experiments.

Although only one membrane of each type was fabricated for this study, the experiments demonstrate reproducibility based on our previous work (Nascimben Santos et al. 2022). In that study, over 50 membranes were fabricated in random order, and a comprehensive statistical analysis using central composite design confirmed that the synthesis protocol is both reproducible and reliable.

### Modification of membranes

The membranes were modified with commercial TiO_2_ (Aeroxide P25, Evonik Industries, Hanau-Wolfgang, Germany; *d* = 25–39 nm), our own BiVO_4_ (following the methodology described in a previous study of our research group (Nascimben Santos et al. 2020)), and commercial CNT (Nanothinx NTX1, Patra, Greece; l ≥ 10 μm; d = 15–35 nm) nanomaterials, while PVP (Sigma-Aldrich®, 40 kDa, Saint Louis, MI, USA) was used as a pore former. All the materials were dried in an oven at 80 °C for 4 h in glass vessels prior to the experiments. The composites of the three nanomaterials (TiO_2_, BiVO_4_, CNT) were prepared by grinding them together in calculated amounts in an agate mortar for 15 min. BiVO_4_ was applied in two different ratios (2 and 20 wt.%), while CNT was applied in 2 wt.%, as it was found to be the most beneficial ratio in a previous study about TiO_2_/CNT-coated membranes (Fekete et al. 2023). Therefore, 100%-TiO_2_, 98%-TiO_2_/2%-BiVO_4_, 98%-TiO_2_/2%-CNT, 80%-TiO_2_/20%-BiVO_4_, and 78%-TiO_2_/20%-BiVO_4_/2%-CNT-composite-modified membranes were prepared. The combinations of the materials and the arrangement of the membranes are presented together with the results in Table 1.

**Table 1:** Composition of the investigated membranes (*N*, neat; *T*, TiO_2_; *B2*, 2 wt.% BiVO_4_; *B20*, 20 wt.% BiVO_4_; *C*, 2wt.% CNT; *-Bl*, blended; *-Co*, coated; *-Gr*, grafted; *-PVP*, 1 wt.% polyvinylpyrrolidone)

Modification method	Membrane type	Ratio of TiO_2_:BiVO_4_:CNT	PVDF	PVP	TiO_2_	BiVO_4_	CNT	NMP
			wt.% (40.0 g total)					
Blended	N-Bl	0:00:00	15	0	0	0	0	85
	T-Bl	100:00:00	15	0	1	0	0	84
	TB2-Bl	98:02:00	15	0	0.98	0.02	0	84
	TC-Bl	98:00:02	15	0	0.98	0	0.02	84
	TB20-Bl	80:20:00	15	0	0.8	0.2	0	84
	TB20C-Bl	78:20:02	15	0	0.78	0.2	0.02	84
	TC-Bl-PVP	98:00:02	15	1	0.98	0	0.02	83
Coated	N-Co	0:00:00	15	0	0	0	0	85
	T-Co	100:00:00	15	0	1	0	0	84
	TB2-Co	98:02:00	15	0	0.98	0.02	0	84
	TC-Co	98:00:02	15	0	0.98	0	0.02	84
	TB20-Co	80:20:00	15	0	0.8	0.2	0	84
	TB20C-Co	78:20:02	15	0	0.78	0.2	0.02	84
	TC-Co-PVP	98:00:02	15	1	0.98	0	0.02	83
Grafted	N-Gr	0:00:00	15	0	0	0	0	85
	T-Gr	100:00:00	15	0	1	0	0	84
	TB2-Gr	98:02:00	15	0	0.98	0.02	0	84
	TC-Gr	98:00:02	15	0	0.98	0	0.02	84
	TB20-Gr	80:20:00	15	0	0.8	0.2	0	84
	TB20C-Gr	78:20:02	15	0	0.78	0.2	0.02	84
	TC-Gr-PVP	98:00:02	15	1	0.98	0	0.02	83

The blended membranes were fabricated following the same method described in our previous study (Nascimben Santos et al. 2022). The additive (PVP) and the nanomaterials were incorporated into the membrane matrix. For the PVP-containing membranes, 1 wt.% of the additive was added to the NMP solvent and ultrasonicated for 2 min (UP200S, Hielscher, Teltow, Germany). Then, nanocomposites containing different ratios of TiO_2_, BiVO_4_, and CNT were added into the solution in 1 wt.% according to Table 1. The suspensions were ultrasonicated again for 2 min before the addition of the dried PVDF polymer. After that, the dope solutions were stirred and ultrasonicated following the same method used for preparing the neat membrane detailed above. Last, the membranes were also cast using the same methodology.

For the fabrication of coated membranes, 1 wt.% amounts of the given nanomaterials were immobilized by physical deposition onto the surface of the previously fabricated neat PVDF membranes. The amount of composite needed was calculated in such a way as to ensure the same quantity was used in all the membranes, making them comparable. A total of 10 mg of the nanomaterial(s) was suspended in 100 mL of 2-propanol and dispersed for 2 min with a high-power ultrasonic homogenizer (200 W, 24 kHz, amplitude = 1, cycle = 100%, Hielscher UP200S, Teltow, Germany) combined with intense magnetic stirring. Then, the suspensions were filtered through the PVDF membranes using a batch-stirred membrane reactor (Millipore, XFUF07601, Burlington, MA, USA), using 0.3 MPa transmembrane pressure. Finally, the membranes were dried in the air at room temperature.

To fabricate the grafted membranes, we followed the steps described in the study of Madaeni et al. (Madaeni et al. 2011). Firstly, 20 g of polymerization solution was fabricated: 20 wt.% acrylic acid monomer (Sigma-Aldrich, anhydrous, 99%, Saint Louis, MI, USA) and 75.35 wt.% water were mixed and stirred for 5 min. Then, 1 wt.% (0.2 g) of the given nanomaterial was added, and the suspension was stirred for 5 more minutes, followed by 10 min of ultrasonication. As the next step, 2.65 wt.% ethylene glycol cross-linker (Scharlab Magyarország Kft., analytical grade, Debrecen, Hungary) and 1 wt.% potassium persulfate initiator (Scharlab Magyarország Kft., analytical grade, Debrecen, Hungary) were added, followed by stirring and ultrasonication for further 10 min. The solution was poured into a glass container, and the PVDF membranes were dipped in it. The system was mixed in a shaking machine (Medline Scientific Limited BS-11, Rotherham, UK) for 30 min at a shaking speed of 80 rpm at room temperature. The membranes were placed on a glass plate and then irradiated with UV-C light (Philips, 2 × 18 W; Pila, Poland) for 30 min. In the end, the grafted membranes were dried for 4 h at 60 °C.

### Membrane characterization

Contact angle measurements (Dataphysics Contact Angle System OCA15Pro, Filder-stadt, Germany) were carried out to characterize the hydrophilicity of the membrane surfaces. A 10 μL volume of ultrapure water was carefully dropped onto the surfaces, and the contact angles formed between the given membrane and the ultrapure water droplet were measured immediately (within 1 s). The measurements were repeated four times, and the results were averaged.

In addition, some of the fabricated membranes were also characterized by field emission scanning electron microscopy (FESEM, Hitachi S-4700 Type II, Krefeld, Germany). The applied acceleration voltage was 10 kV. The membranes were dried and coated with gold before analysis.

The photocatalytic activities of the membranes were determined by the photocatalytic decolorization of 100 mL of methyl orange (MO) solution (Sigma-Aldrich, Chemie GmbH, Saint Louis, MI, USA; 10^–5^ M = 3.27 mg L^–1^) in a photoreactor equipped with a UV-A lamp (Sylvania F18W/BLB-T8, Blacklight-Blue, 2 × 18W, Erlangen, Germany). First, the membrane submerged in the dye solution was kept in the dark overnight to reach *steady-state* adsorption. After stabilization, the UV lamp was turned on (time 0), and samples were taken at regular intervals (time t) during the 24-h-long experiments. The absorbances were recorded with a spectrophotometer (Biochrom Biowave II + , Cambridge, UK) at *λ* = 466 nm. The same experiments were also carried out using visible light (Tungsram FT8-18W-840; 2 × 18 W, Nagykanizsa, Hungary) and simulated solar light (Sylvania 18W-Activa172; 2 × 18 W, Erlangen, Germany).

To describe the photocatalytic efficiency, we calculated the decolorization rate (ϵ) by the following equation:

1 $$\epsilon =\frac{{Abs}_{0}-{Abs}_{t}}{{Abs}_{0}}\times 100\ [\%]$$

where Abs_0_ and Abs_t_ are the absorbances of the dye solution measured when the lamp was turned on (time 0) and after a certain time of irradiation (time *t*), respectively.

It is known that the decolorization of methyl orange with a low initial concentration follows pseudo-first-order kinetics; therefore, it can be described by the Langmuir–Hinshelwood kinetics law (Neghi et al. 2019; Hir et al. 2017; Chen et al. 2018; Barka et al. 2008; Benhabiles et al. 2019), using the following equation:

2 $$\mathit{ln}\left(\frac{{Abs}_{t}}{{Abs}_{0}}\right)= - k \times t$$

where Abs_0_ and Abs_t_ are the absorbances of the dye solution measured after reaching the *steady state* (time 0) and after a certain time of irradiation (time *t*), respectively, *k*_0_ is the initial apparent first-order kinetics rate constant expressed by the slope of the graph (h^–1^), and *t* is the reaction time (h). For the calculation of kinetics, we considered only the first 8 h of the experiment where the suppression of the photocatalytic decolorization by the oxidation by-products is less significant. Then, the initial pseudo-first-order reaction rate (*r*_0_) was calculated considering the initial concentration (*C*_0_) and *k*_0_:

3 $${r}_{0}= {{k}_{0} \times C}_{0} \ [mg {L}^{-1} {h}^{-1}]$$

### Preparation of oil-in-water emulsions

The used crude oil was provided by a South Hungarian oil producer company. The preparation of oil emulsions (*c*_oil_ = 100 mg L^–1^) was carried out by adding 1 wt.% crude oil to ultrapure water followed by rigorous mixing (Skil F0151415AC, Teuchern, Germany; 35000 rpm, 30 s) and 10 min of ultrasonic homogenization (Hielscher UP200S, Teltow, Germany) at 24 kHz frequency (using maximal amplitude and cycle). A constant temperature of 25 °C was set by circulating water in the thermostat jacket of the double-walled glass reactor. The oil droplets of the prepared oil-in-water emulsion had a diameter of 50–1500 nm with an average droplet size of ~ 500 nm (determined by dynamic light scattering measurements).

### Filtration experiments

All the membranes were soaked in pure water for 3 h before starting the filtration process. First, ultrapure water was filtered in a batch-stirred dead-end membrane reactor (Millipore XFUF07601, Burlington, MA, USA), which was equipped with the given wet membrane (active filtration area: 36.2 cm^2^). The water was filtered by applying 100 kPa transmembrane pressure and 250 rpm stirring for 30 min to reach a steady state and ensure the compaction of the membranes. Then, the pure water flux (J_w_) was recorded with a computer-controlled scale at predetermined intervals.

The fluxes (J) were calculated as follows:

4 $$J=\frac{\Delta V}{A\times \Delta t} [L {m}^{-2} {h}^{-1}]$$

where *J* is the permeation flux (L m^−2^ h^−1^), Δ*V* is the permeate volume (L), *A* is the effective membrane area (m^2^), and Δ*t* is the sampling time interval (h).

After the determination of pure water flux, 250 mL of oil emulsion was added to the reactor and filtered until 200 mL of permeate was obtained (volume reduction ratio: VRR = 250/(250–200) = 5). During this, the flux was continuously measured (J_oil_). Purification efficiencies were additionally determined by measuring the chemical oxygen demand (COD) using a standard potassium-dichromate oxidation-based method, with standard test tubes (Hanna Instruments, Szeged, Hungary). The digestions were carried out in a COD digester (Lovibond, ET 108, Dortmund, Germany) for 2 h at 150 °C, and the COD values were measured with a COD photometer (Lovibond COD Vario, Dortmund, Germany). After achieving the VRR of 5, we rinsed the membrane with pure water and measured the water flux again to check its recoverability (*J*_after_).

Filtration resistances represent factors that impede flow through membranes; therefore, they are crucial to evaluate the performance of the membranes (Kasemset et al. 2013; Wu et al. 2018; Kovács et al. 2018; Gokulakrishnan et al. 2021). The resistance of the membrane (*R*_M_) is characteristic of its material and structure, which was calculated as

5 $${R}_{M}=\frac{\Delta p}{{J}_{W}\times{\eta }_{W}} \left[{m}^{-1}\right]$$

where Δ*p* is the applied transmembrane pressure [Pa], *η*_*W*_ is the viscosity of the water [Pa s], and *J*_*W*_ is the pure water flux of the membrane.

Irreversible filtration resistance (*R*_Irrev_) originates from contaminants irreversibly attached to the pores and surface, which cannot be easily removed. *R*_Irrev_ was determined according to the water flux (*J*_after_) measured after the filtration of the oil emulsion and the purification of the used membrane (carried out by intense rinsing with pure water):

6 $${R}_{\mathit{Irrev}}=\frac{\Delta p}{{J}_{after }\times{\eta }_{W}}-{R}_{M} \left[{m}^{-1}\right]$$

Reversible filtration resistance (*R*_Rev_)—caused by non-attached, easily removable oil droplets and concentration polarization—was calculated as

7 $${R}_{\mathit{Rev}}=\frac{\Delta p}{{J}_{oil }\times{ \eta }_{oil}}-{R}_{Irrev}-{R}_{M} \left[{m}^{-1}\right]$$

where *η*_oil_ is the viscosity of the emulsion and *J*_oil_ is the flux of the oil emulsion at VRR = 5.

Total filtration resistance (RT) can be calculated as the sum of the previously detailed resistances, in accordance with the resistance-in-series model:

$${R}_{T}= {R}_{M}+ {R}_{Irrev}+ {R}_{Rev} \left[{m}^{-1}\right]$$

To evaluate the fouling resistance of the membranes in different conditions, we also calculated the flux decay ratios (FDRs) and flux recovery ratios (FRRs):

8 $$\mathit{FDR}= \frac{Jw-Joil}{Jw}\times 100\ [\%]$$

9 $$\mathit{FRR}= \frac{{J}_{after}}{Jw}\times 100\ [\%]$$

## Results and discussion

### Membrane characterization

**Fig. 1:**
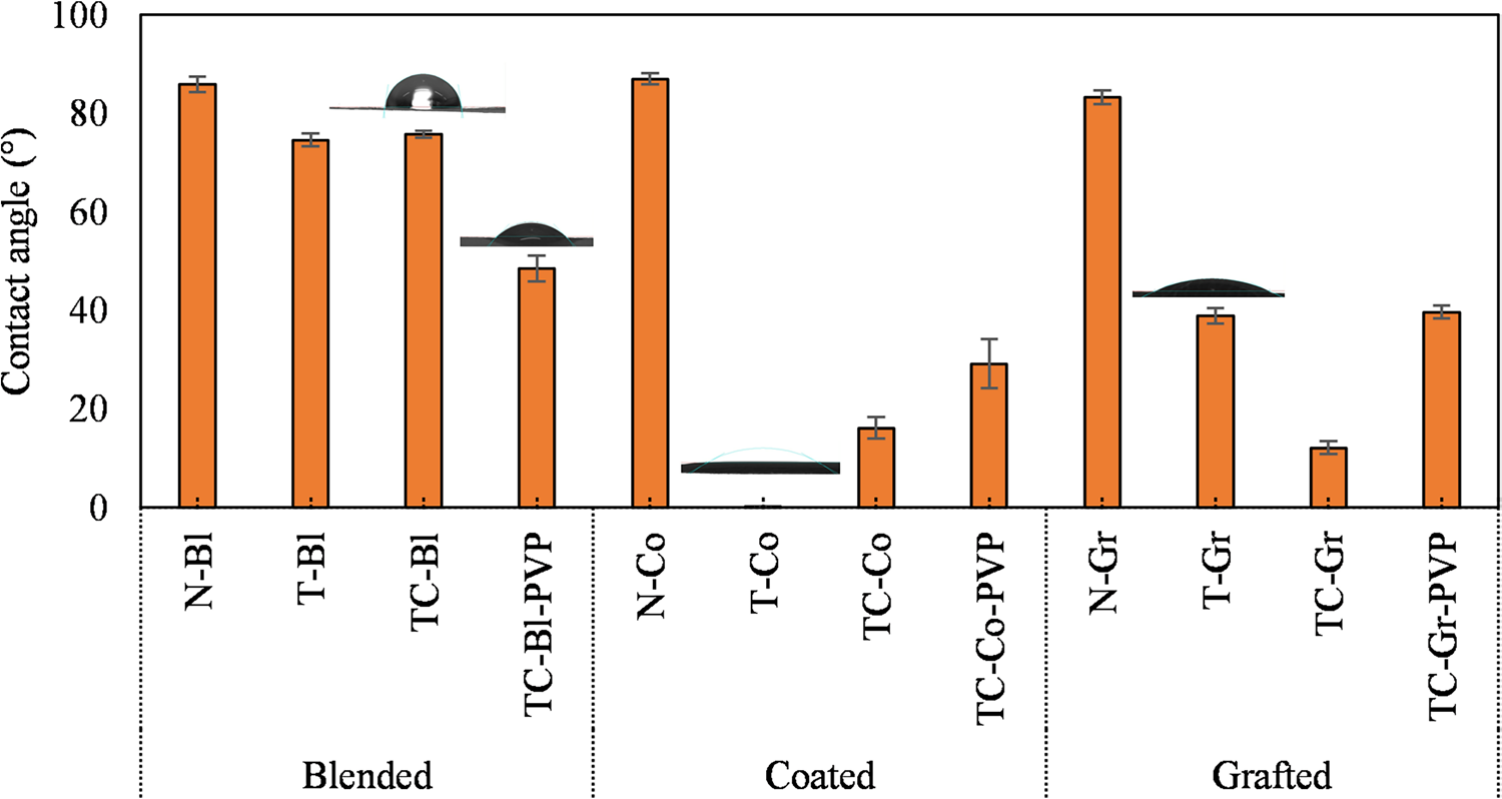
Water contact angles of the investigated membranes (N, neat; T, TiO_2_; C, 2wt.% CNT; PVP, 1 wt.% polyvinylpyrrolidone; -Bl, blended; -Co, coated; -Gr, grafted)

Figure 1 shows the main results of the contact angle measurements that are also presented in Table 2, demonstrating that all nanocomposite membranes exhibited lower contact angles compared with the neat ones. The contact angles of the neat membranes were between 80 and 90°, which is in good accordance with other studies (Acarer et al. 2021; Zhang et al. 2022; Hudaib et al. 2022). In general, the coated membranes resulted in the lowest contact angles, but it is important to note that this outcome does not necessarily guarantee improved filtration performance. Other factors come into play, such as the potential clogging of nanoparticles within the membrane pores or the formation of a compact cake layer containing the nanoparticles. These factors can reduce the fluxes of membranes. A more detailed and comprehensive analysis of these effects will be presented and explained in the following section.

**Table 2:** Contact angle values and decolorization of methyl orange (C_0_ = 10^–5^ M, ~ 3.27 mg L^–1^). Initial apparent rate constants k_0_ (h^–1^) were calculated from the slope of ln(Abs/Abs_0_) versus time (until 8 h of irradiation), *R*^2^ is the correlation coefficient of the generated curve, ϵ_8_ is the decolorization efficiency obtained after 8 h (%), and *r* is the initial reaction rate (mg L^–1^ h^–1^) (*N*, neat; *T*, TiO_2_; *B2*, 2 wt.% BiVO_4_; *B20*, 20 wt.% BiVO_4_; *C*, 2wt.% CNT; *-PVP*, 1 wt.% polyvinylpyrrolidone; *-Bl*, blended; *-Co*, coated; *-Gr*, grafted)

Modification method	Membrane name	Ratio of TiO_2_:BiVO_4_:CNT	Contact angle (°)	*r* (mg L^–1^ h^–1^)	*k*_0_ (h^–1^)	*R*^2^	ϵ_8_ (%)
Blended	N-Bl	0:00:00	85.1	~ 0.0	~ 0.0	0.605	0.0
	T-Bl	100:00:00	75.4	0.106	− 0.032	0.976	23.1
	TB2-Bl	98:02:00	79.4	0.071	− 0.022	0.819	17.2
	TC-Bl	98:00:02	75.1	0.316	− 0.097	0.998	52.6
	TB20-Bl	80:20:00	71.6	~ 0.0	~ 0.0	0.579	0.0
	TB20C-Bl	78:20:02	78.9	~ 0.0	~ 0.0	0.653	0.0
	TC-Bl-PVP	98:00:02	44.8	0.356	− 0.109	0.995	56.0
Coated	N-Co	0:00:00	86.7	~ 0.0	~ 0.0	0.717	0.0
	T-Co	100:00:00	~ 0.0	0.380	− 0.116	0.995	60.5
	TB2-Co	98:02:00	~ 0.0	0.346	− 0.106	0.987	58.3
	TC-Co	98:00:02	17.4	0.316	− 0.097	0.988	55.5
	TB20-Co	80:20:00	~ 0.0	~ 0.0	~ 0.0	0.689	4.7
	TB20C-Co	78:20:02	6.5	~ 0.0	~ 0.0	0.906	6.9
	TC-Co-PVP	98:00:02	23.6	0.911	− 0.279	0.995	88.4
Grafted	N-Gr	0:00:00	81.5	~ 0.0	~ 0.0	0.925	22.2
	T-Gr	100:00:00	36.8	0.407	− 0.124	0.994	62.3
	TB2-Gr	98:02:00	47.1	0.209	− 0.064	0.988	46.6
	TC-Gr	98:00:02	11.1	0.623	− 0.191	0.988	74.9
	TB20-Gr	80:20:00	48.4	0.220	− 0.067	0.998	46.9
	TB20C-Gr	78:20:02	57.4	0.216	− 0.066	0.992	57.1
	TC-Gr-PVP	98:00:02	40.2	0.677	− 0.207	0.994	79.2

In general, the grafted membrane had a lower contact angle in comparison with the blended membranes. This is certainly related to the higher nanoparticle content on the surface of the grafted membranes (while in the blended membranes, the nanoparticles are spread in the whole membrane matrix). This result can also be attributed to the introduction of carboxyl groups with the acrylic acid used in the grafting method, which enhanced the hydrophilicity of the membranes, promoting the wetting of the surface, as reported in several studies (Madaeni et al. 2011; Liang et al. 2013). However, interestingly, the addition of PVP reduced the contact angles for the blended membranes but increased them for the grafted membranes. This can be explained by the interaction between the grafted acrylic acid and PVP chains, which can affect the overall surface energy and wettability of the membranes. The presence of PVP can hinder the interaction of water with the grafted carboxyl groups, causing a reduced wetting effect (Sohail et al. 2014).

Figure 1 shows that the addition of CNT to TiO_2_ increases the contact angle for the coated membrane, indicating a reduction in hydrophilicity. This outcome was expected as CNT is a hydrophobic nanomaterial, which can notably reduce the superhydrophilic nature of the pure TiO_2_-coated surface. However, for grafted membranes, the addition of CNT enhanced the hydrophilicity (i.e., reduced the contact angle). This can be attributed to the fact that acrylic acid was introduced to the solution during membrane fabrication, possibly resulting in the functionalization of CNT. Consequently, this functionalization process can help to reduce the hydrophobicity of the material and, therefore, the contact angle, as presented in other studies (Zhao et al. 2019; Gao et al. 2020; Hudaib et al. 2022; Yu et al. 2020).

**Fig. 2:**
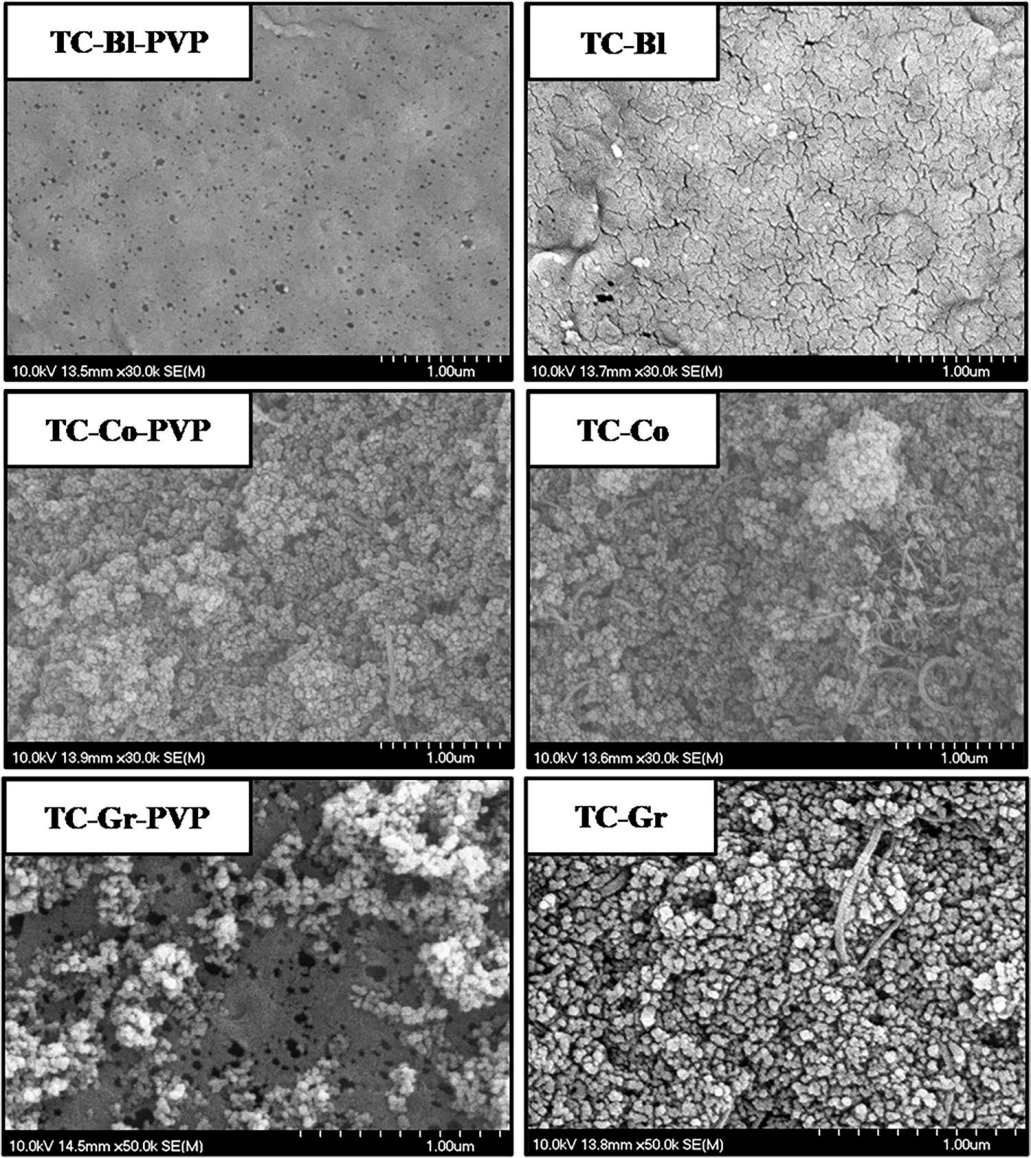
Scanning electron microscopy images of the TiO_2_–CNT-containing membranes (T, TiO_2_; C, 2 wt.% CNT; -PVP, 1 wt.% polyvinylpyrrolidone; -Bl, blended; -Co, coated; -Gr, grafted)

The scanning electron microscopy images of the TiO_2_–CNT-containing membranes (Fig. 2) show that bigger pores were formed when PVP was used, which is a pore former (Yoo et al. 2004; Zhou and Xi 2007; Nascimben Santos et al. 2022). The presence of CNTs could be observed in the membranes, confirming their successful addition. For the blended membranes, detecting the nanotubes is challenging because they are not only on the surface of the membrane but also in the bulk. For the coated membrane, some agglomerates of CNTs were also formed due to their high density on the membrane surface.

### Photocatalytic activity

**Fig. 3:**
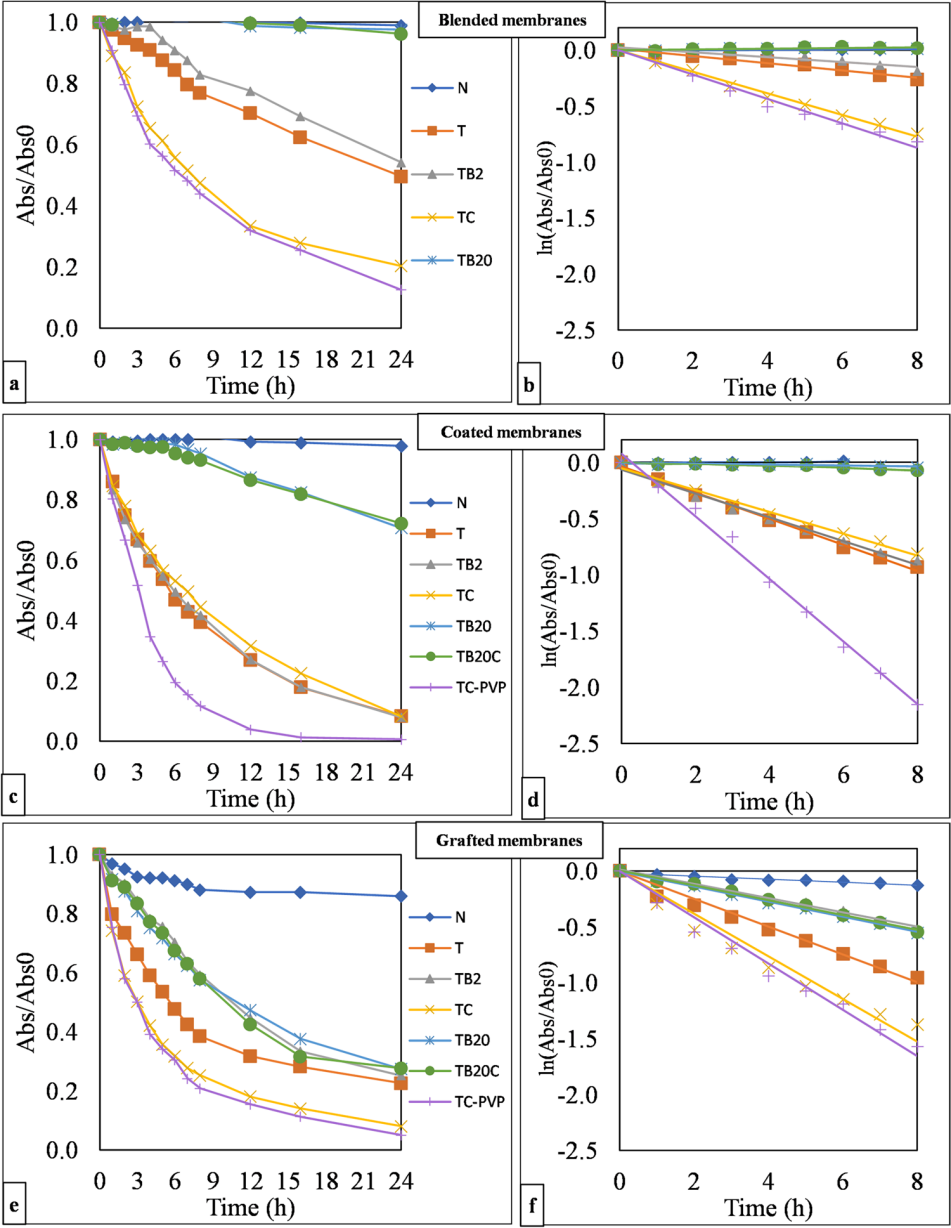
Absorbance of methyl orange determined for all fabricated membranes under UV irradiation for 24 h (**a**, **c**, and **e**) and initial apparent rate constants (*k*) obtained by plotting the ln(Abs/Abs_0_) of methyl orange as a function of time up to 8 h of the experiment (**b**, **d**, and **f**); N, neat; T, TiO_2_; B2, 2 wt.% BiVO_4_; B20, 20 wt.% BiVO_4_; C, 2wt.% CNT; PVP, 1 wt.% polyvinylpyrrolidone

Figure 3 and Table 2 demonstrate that the incorporation of PVP had a positive impact on the photocatalytic activity. The PVP-modified membranes presented the highest photocatalytic activity in all three series (blended, coated, and grafted membranes), especially in the case of the coated membranes. The enhancement of the photocatalytic decolorization can be attributed to the ability of PVP to increase the membrane pores, increasing the irradiated surface area available for nanoparticle immobilization and enabling light to penetrate even the innermost regions of the membrane (Hyun et al. 2004; Nascimben Santos et al. 2022; Mahdavi et al. 2020; Pagidi et al. 2014). As a result, photocatalytic nanoparticles dispersed throughout the bulk of the membrane can also be effectively accessed, leading to improved photocatalytic performance.

In addition, combining CNTs with TiO_2_ results in a significantly better performance than using only TiO_2_ for blended and grafted membranes. CNTs have good electrical conductivity and a large surface area, facilitating the transfer of photoexcited electrons from TiO_2_ to CNT. This electron transfer contributes to the suppression of recombination, leading to enhanced photocatalytic activity. In addition, CNTs have a particularly high adsorption capacity for organic compounds such as the dye we used: methyl orange (Pagidi et al. 2014; Zhao et al. 2019; Veréb et al. 2022). The adsorption of methyl orange increased its concentration on the photocatalyst surface, which could also contribute to the higher photocatalytic efficiency.

It is important to note that the pH of the methyl orange solution was ≅ 5.3–5.6, which is its natural pH. At this pH, the photocatalyst surface is positively charged, while methyl orange carries a negative charge, resulting in attractive forces between them, which can ensure high dye removal efficiency, as demonstrated in our previous research (Nascimben Santos et al. 2022).

The TiO_2_/BiVO_4_ membranes (TB2) exhibited photocatalytic activity similar to that of pure TiO_2_ under UV light, meaning that 2% BiVO_4_ addition did not improve the photocatalytic efficiency. It is well-known that TiO_2_ has high photoactivity when excited below 400 nm wavelengths (Rahimpour et al. 2008; Kovács et al. 2018; Nascimbén Santos et al. 2020) due to its higher quantum efficiency in the UV range (Veréb et al. 2022), meaning that TiO_2_ was mainly responsible for the degradation. It is important to note that even the higher BiVO_4_ concentration (20% in TB20 and TB20C) did not yield higher photocatalytic efficiencies in these UV experiments. This outcome can be attributed to the fact that these membranes contained fewer of the much more UV-active TiO_2_ particles, and they were more significantly covered by the much bigger BiVO_4_ nanoparticles, leading to less activated TiO_2_ on the surface, which could negatively affect UV-induced photocatalytic reactions.

Among the three different modification methods, the coating approach resulted in the highest photocatalytic activity, evident from its steeper curve slope (*k*). The second-best photocatalytic efficiencies were observed for membranes prepared by grafting. This can be attributed to the fact that both coating and grafting methods primarily modify the membrane surfaces, ensuring the availability and accessibility of nanoparticles for light activation. Coating, which results in a uniform layer of photocatalytic material, promotes efficient light absorption and effective interaction with pollutants, resulting in enhanced photocatalytic activity. Grafting, while slightly less efficient, still leads to improved activity compared with the activities measured for the blended (and unmodified) membranes. In contrast, blending places the nanoparticles within the bulk of the membrane, resulting in their reduced availability on the surface and, therefore, reduced photocatalytic activity (Pendergast and Hoek 2011; Van der Bruggen 2009; Miller et al. 2016; Nascimbén Santos et al. 2020; Gokulakrishnan et al. 2021).

Intriguingly, when all three components (TiO_2_, BiVO_4_, and CNT) are combined, the overall photocatalytic activity appears to be lower compared with using TiO_2_ with either BiVO_4_ or CNT individually. More experiments need to be carried out to evaluate the synergetic effect of TiO_2_, BiVO_4_, and CNT when used simultaneously. The components can compete with each other or even increase the possibility of agglomeration, resulting in lower surface area, lower accessibility, and therefore, lower photocatalytic efficiency. For a better understanding, it is crucial to evaluate the optimal composition and ratio in composite systems.

**Fig. 4:**
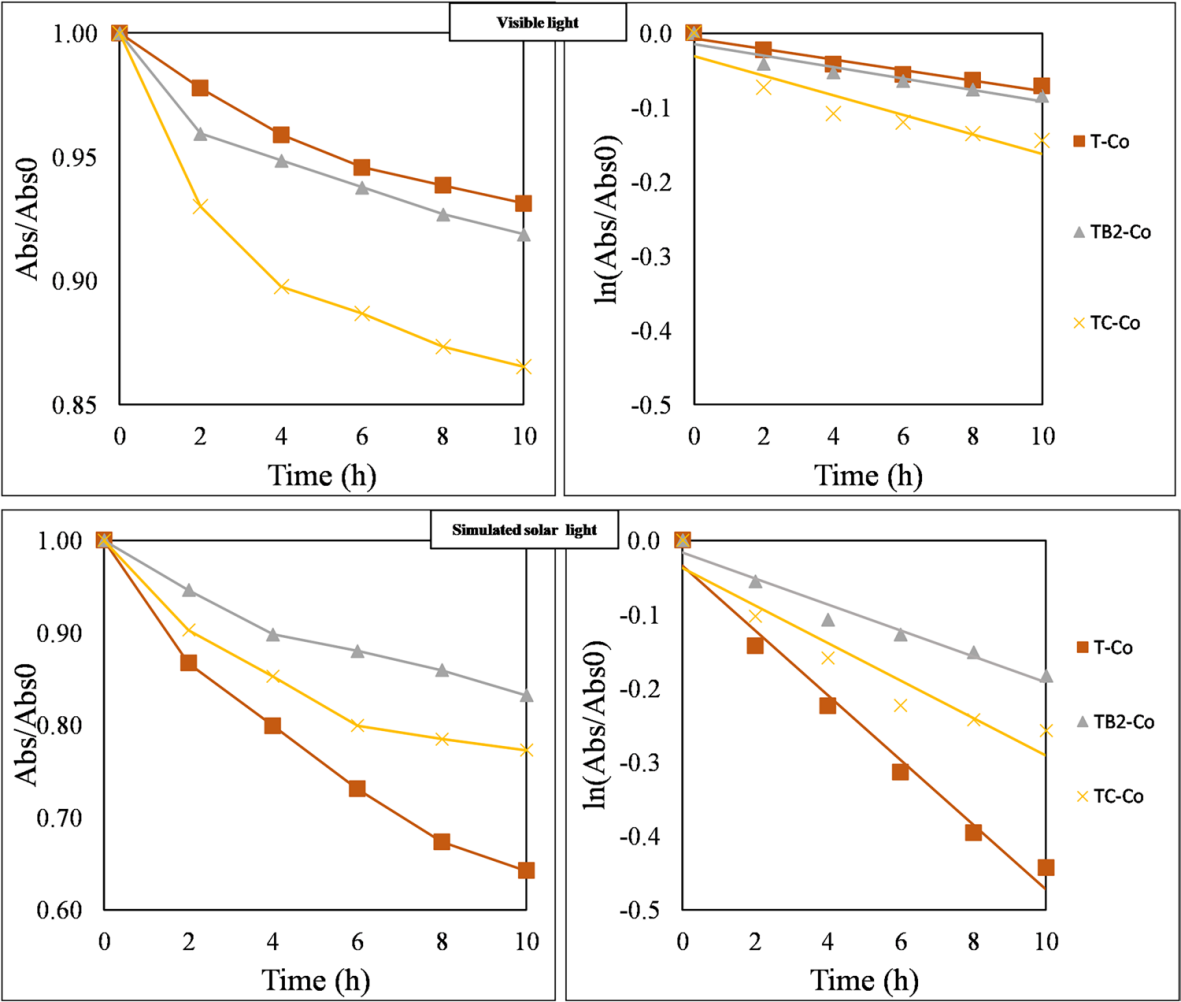
Absorbance of methyl orange for all fabricated membranes during visible light and simulated solar irradiations for 10 h and initial apparent rate constant (*k*_0_) calculation plotting the ln(Abs/Abs_0_) (T, TiO_2_; B2, 2 wt.% BiVO_4_; C, 2wt.% CNT; -Co, coated membranes)

The coated membranes had the highest decolorization efficiency due to their highest photocatalytically active surface areas. Therefore, their photocatalytic activity was evaluated under visible light and simulated solar irradiations too (Fig. 4). Even though BiVO_4_ was not beneficial for UV-photocatalysis, the 2 wt.% BiVO_4_-containing membranes demonstrated superior performance under visible light irradiation (*k*_0_ = –0.008) than the ones containing only TiO_2_ (*k*_0_ = –0.007) due to the narrower band gap of BiVO_4_ (Sisay et al. 2023; Wetchakun et al. 2015; Nascimben Santos et al. 2020; Song et al. 2017). The TiO_2_-CNT membranes had the greatest photocatalytic activity under visible light (*k*_0_ = –0.013), demonstrating the capability of CNTs to facilitate charge separation and suppress electron–hole recombination (Sisay et al. 2023; Veréb et al. 2019; Fekete et al. 2023; Liu et al. 2017; Venkatesh et al. 2020). On the other hand, the TiO_2_-coated membranes still had better performance for the simulated solar experiments (*k*_0_ = –0.044) when compared with the 2 wt.% BiVO_4_- (*k*_0_ = –0.017) and CNT- (*k*_0_ = − 0.025) containing composite-coated membranes, due to their high efficiency under UV light. All the results of the photocatalytic experiments are presented in the Appendix.

### Filtration experiments

**Fig. 5:**
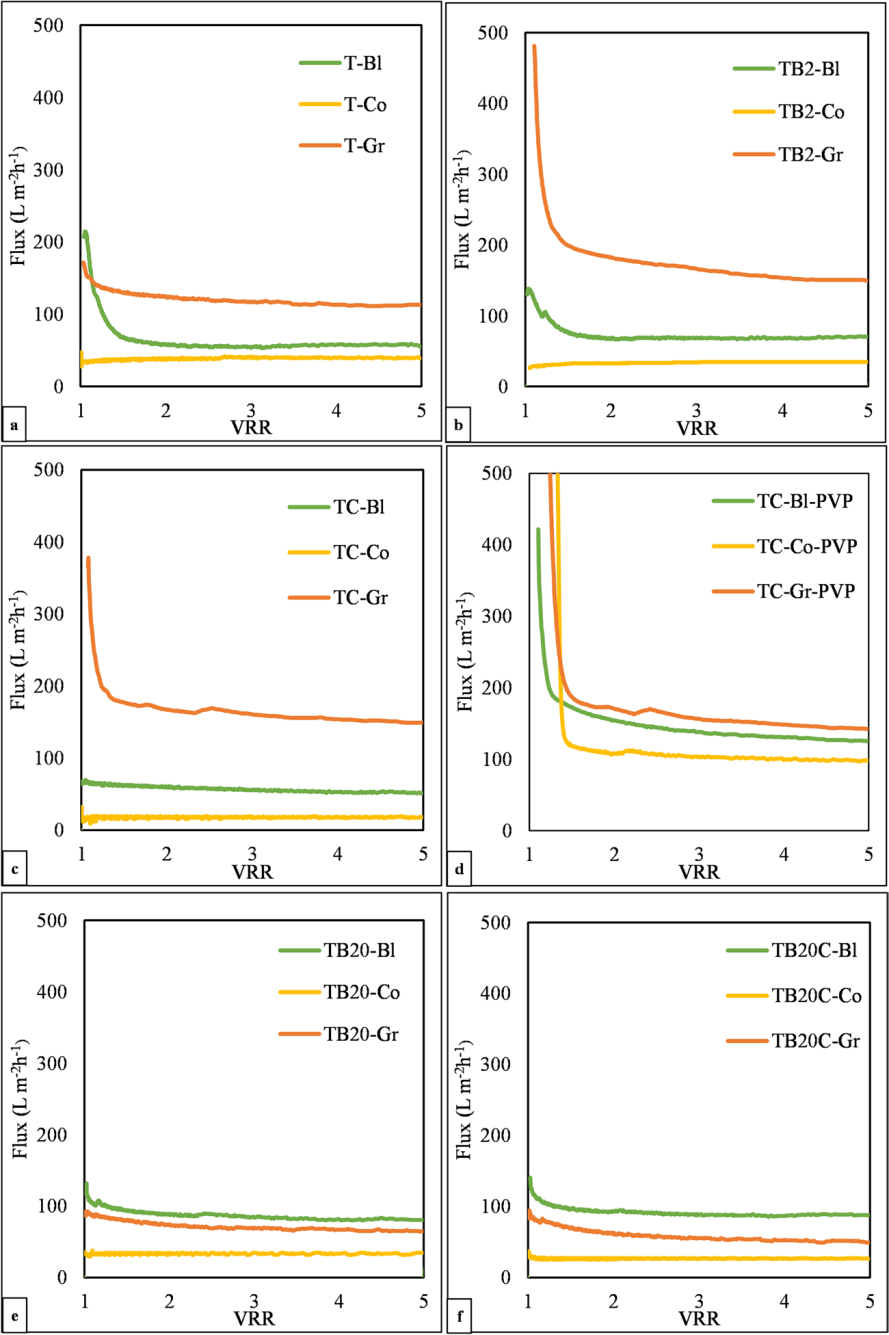
Flux curves measured during the filtration of the oil emulsions with the membranes modified with 100 wt.% TiO_2 _(**a**), 98 wt.% TiO_2_ and 2 wt.% BiVO_4_ (**b**), 98 wt.% TiO_2_ and 2 wt.% CNT (**c**), 98 wt.% TiO_2_ and 2 wt.% CNT with 1 wt.% PVP (**d**), 80 wt.% TiO_2_ and 20 wt.% BiVO_4_(**e**) and with 78 wt.% TiO_2_, 20 wt.% BiVO_4_ and 2 wt.% CNT (**f**) (-Bl, blended; -Co, coated; -Gr, grafted; T, TiO_2_; B2, 2 wt.% BiVO_4_; B20, 20 wt.% BiVO_4_; C, 2wt.% CNT; PVP, 1 wt.% polyvinylpyrrolidone)

The main results of the filtration experiments are presented in Table 3 and Fig. 5. Initially, it is worth mentioning that all the fabricated membranes presented more than 98% efficiency in removing the oil from the oil-in-water emulsions, evaluated based on the COD of the permeates.

**Table 3:** Composition of the membranes and the results of the filtration experiments. *J*_*w*_ is the flux of water after compaction, *J*_oil_ is the flux of the oil emulsion at VRR = 5, *J*_after_ is the water flux after cleansing the membrane with ultrapure water, FDR is the flux decay ratio, and FRR is the flux recovery ratio (N, neat; T, TiO_2_; B2, 2 wt.% BiVO_4_; B20, 20 wt.% BiVO_4_; C, 2wt.% CNT; -PVP, 1 wt.% polyvinylpyrrolidone; -Bl, blended; -Co, coated; -Gr, grafted)

Modification method		Membrane name	Ratio of TiO_2_:BiVO_4_:CNT (wt.%)	Fluxes (L m^–2^ h^–1^)			FDR (%)	FRR (%)	Filtration resistances (m^–1^)		
				*J*_*w*_	*J*_oil_	*J*_after_			Membrane resistance	Reversible resistance	Irreversible resistance
Blended	T-Bl		100:00:00	140.6	55.2	57.9	60.8	41.2	2.6E + 06	7.3E + 09	3.7E + 06
	TB2-Bl		98:02:00	102.0	70.7	71.1	30.7	69.7	3.5E + 06	5.7E + 09	1.5E + 06
	TC-Bl		98:00:02	89.6	50.9	67.46	43.2	75.2	4.0E + 06	7.9E + 09	1.3E + 06
	TB20-Bl		80:20:00	105.8	78.00	80.5	26.3	76.1	3.4E + 06	5.2E + 09	1.1E + 06
	TB20C-Bl		78:20:02	130.5	87.7	89.2	32.8	68.3	2.8E + 06	4.6E + 09	1.3E + 06
	TC-Bl-PVP		98:00:02	2,165.7	124.9	642.3	94.2	29.7	8.9E + 04	6.6E + 09	5.9E + 05
Coated	T-Co		100:00:00	44.8	38.6	42.0	13.8	93.7	8.0E + 06	1.0E + 10	5.4E + 05
	TB2-Co		98:02:00	42.2	34.1	40.1	19.2	94.9	8.5E + 06	1.2E + 10	4.6E + 05
	TC-Co		98:00:02	20.5	18.2	19.8	11.3	96.7	1.8E + 07	2.2E + 10	5.9E + 05
	TB20-Co		80:20:00	42.7	34.3	41.2	19.6	96.4	8.4E + 06	1.2E + 10	3.1E + 05
	TB20C-Co		78:20:02	43.1	30.1	35.1	30.1	81.5	8.3E + 06	1.3E + 10	1.9E + 06
	TC-Co-PVP		98:00:02	1948.2	97.2	976.8	95.0	50.1	1.4E + 05	2.9E + 09	3.9E + 05
Grafted	T-Gr		100:00:00	193.1	110.2	118.4	42.9	61.3	1.9E + 06	3.7E + 09	1.2E + 06
	TB2-Gr		98:02:00	251.5	146.0	237.0	41.9	94.3	1.4E + 06	2.8E + 09	8.7E + 04
	TC-Gr		98:00:02	178.9	147.8	158.8	17.4	88.8	2.0E + 06	2.7E + 09	2.5E + 05
	TB20-Gr		80:20:00	91.9	63.6	64.0	30.8	69.6	3.9E + 06	6.4E + 09	1.7E + 06
	TB20C-Gr		78:20:02	93.5	49.6	49.9	47.0	53.3	3.8E + 06	8.2E + 09	3.4E + 06
	TC-Gr-PVP		98:00:02	2630.5	141.8	679.2	94.6	25.8	1.2E + 05	6.9E + 09	8.3E + 05

During the filtration experiments conducted using membranes modified with TiO_2_ (Fig. 5a), TiO_2_/BiVO_4_(2%) composite (Fig. 5b), or TiO_2_/CNT(2%) composite (Fig. 5c), the grafted membranes exhibited superior filtration performance (2–7 times higher fluxes, up to 148 L m^–2^ h^–1^) compared with the coated and blended membranes. The grafted membranes, with the chemically bonded nanoparticles, also showed improved retention and nanoparticle stability, which is in agreement with the literature (Nielsen et al. 2023; Liang et al. 2013; Gokulakrishnan et al. 2021).

On the one hand, the utilization of 20% of BiVO_4_ in the TiO_2_/BiVO_4_ composite (Fig. 5e, f) resulted in lower fluxes for the grafted membranes (64 and 50 L m^–2^ h^–1^ for TB20-Gr and TB20C-Gr, respectively), originating mainly from the significantly lower water fluxes of the clean membranes (92 and 94 L m^–2^ h^–1^ for TB20-Gr and TB20C-Gr, respectively). On the other hand, significant enhancements were observed in the case of the blended membranes (78 and 88 L m^–2^ h^–1^ for TB20-Bl and TB20C-Bl, respectively). This interesting phenomenon occurred because of the increased number of BiVO_4_ nanoparticles mixed within the membrane matrix and not on the surface of the membrane. The higher dispersion, larger surface area, and improved accessibility of the hydrophilic nanoparticles within the membrane matrix of the blended membranes enhanced their filtration performance, leading to higher fluxes.

The experiments were also carried out with the PVP-containing TiO_2_/CNT membranes (Fig. 5d). The results showed a significant improvement at the beginning of the filtration, with fluxes as high as 2,000 L m^–2^ h^–1^. This result was expected due to the pore size enhancement, allowing greater permeability and improved initial flux (Nascimben Santos et al. 2022; Tofighy et al. 2021; Back et al. 2019). The coated membranes benefited from larger pore sizes, which reduced cake layer formation and enhanced water permeability. Moreover, the dispersion of nanoparticles within the membrane matrix was improved in the blended membranes, resulting in enhanced accessibility and better performance. These factors explain why we observed higher flux enhancements for the coated and blended membranes, highlighting the importance of pore size and nanoparticle dispersion to membrane performance. In the case of grafting, further flux enhancement was not achieved by PVP addition (Fig. 5c, d). This outcome is in agreement with the contact angle results, where PVP addition increased the contact angles of the grafted membranes, which was explained by the interaction between the grafted acrylic acid and PVP (Sohail et al. 2014). Within the TC-PVP series, even though PVP addition did not enhance the flux (142 L m^–2^ h^–1^), the grafted membrane still had the best performance when compared with coated (97 L m^–2^ h^–1^) and blended membranes (125 L m^–2^ h^–1^).

Finally, the resistances of the membranes were calculated to assess their lifespan and the recoverability of flux after cleaning. Based on Table 3, the coated membranes exhibited lower water fluxes due to higher intrinsic membrane resistances. For the coated membranes, caution must be exercised during membrane cleaning as the coated layer is physically deposited and susceptible to leaching. Outstandingly, the addition of PVP considerably reduced all resistances in the coated membranes, which can be attributed to the bigger pore sizes that prevent wastewater clogging within the material.

The addition of PVP yielded similar results in terms of resistance reduction for both the blended and grafted membranes. Consequently, the inclusion of PVP led to higher fluxes in all the modified membranes and lower resistances, highlighting its effectiveness in improving the overall membrane performance (Hyun et al. 2004; Back et al. 2019; Nascimben Santos et al. 2022; Tofighy et al. 2021). Indeed, even though the FDR is higher than 94% for all the PVP-containing membranes while the FRR is lower than 50%, the final recovered fluxes of these membranes are still much higher than those of all the other membranes: the least effective PVP-containing membranes exhibited a flux surpassing 600 L m^–2^ h^–1^, whereas the second-best final flux for membranes without PVP was less than 240 L m^–2^ h^–1^. Therefore, the addition of PVP to the membranes offers significant advantages, including enhanced cleaning efficiency, reduced time and effort required for membrane maintenance, and a higher total volume of filtered wastewater at the same time.

The addition of 2% BiVO_4_ or CNT to the blended and grafted membranes resulted in improved membrane performance, particularly in terms of enhanced washability indicated by the lower irreversible resistance and enhanced flux recovery ratios. This improvement can be attributed to the fact that the presence of BiVO_4_ or CNT in low concentrations altered the surface, making it more hydrophilic and reducing its tendency to foul.

## Conclusions

In this study, the addition of CNT and BiVO_4_ to TiO_2_-PVDF membranes was evaluated based on their photocatalytic efficiencies and filtration performances using three different methods (blending, coating, and grafting). The effects of PVP (a pore former) addition were also investigated.

Regarding photocatalytic activity, the addition of CNT to TiO_2_ resulted in improved performance, as evidenced by the enhanced decolorization of methyl orange under both UV and visible light irradiations for grafted and blended membranes. The addition of 2 wt.% BiVO_4_ did not lead to the same level of improvement in the UV experiments; however, the membranes had slightly better performance under visible light in comparison with the performance of TiO_2_-membranes. Nevertheless, the addition of more BiVO_4_ (20 wt.%) did not result in better photocatalytic activity for any of the studied membranes.

In terms of filtration performance, the grafted membranes containing TiO_2_ and 2 wt.% BiVO_4_ or CNT and 1 wt.% PVP exhibited superior performance, achieving higher steady flux compared to the coated and blended membranes. Despite the high photocatalytic activity of the coated membranes resulting from enhanced nanoparticle accessibility on the membrane surface, the filtration experiments of these membranes presented the lowest fluxes.

The addition of PVP improved the photocatalytic efficiency. Although it significantly reduced the flux recovery ratios across all three modification methods, it still resulted in outstanding fluxes during the filtration of emulsions and after the surface cleaning process.

Our findings highlight the importance of nanoparticle selection and modification methods in optimizing the photocatalytic and filtration performance of membranes. Overall, the combination of CNT addition and the utilization of grafting modification proved to be the most beneficial in enhancing the performance of PVDF-TiO_2_ membranes. The PVDF-TiO_2_/CNT_2%_-grafted membrane provided relatively high photocatalytic efficiency (the third highest: only the grafted PVDF-TiO_2_/CNT_2%_/PVP and coated PVDF-TiO_2_/CNT_2%_/PVP membranes had higher photocatalytic efficiency) and the best filtration performance (high flux and flux recovery ratios were achieved with this membrane).

Further research is required to explore novel grafting modification techniques and to develop advanced membranes with satisfactory efficiency for various applications.

## Data Availability

Data are contained within the article.

## Appendix. Absorbance values obtained during photocatalytic experiments using methyl orange under UV, visible, and simulated solar irradiations.

**Table:**

Time (h)	Absorbance of membranes under UV light																				
	Blended							Coated							Grafted						
	N-Bl	T-Bl	TB2-Bl	TC-Bl	TB20-Bl	TB20C-Bl	TC-PVP-Bl	N-Co	T-Co	TB2-Co	TC-Co	TB20-Co	TB20C-Co	TC-PVP-Co	N-Gr	T-Gr	TB2-Gr	TC-Gr	TB20-Gr	TB20C-Gr	TC-PVP-Gr
0	0.352	0.352	0.361	0.361	0.354	0.354	0.353	0.352	0.352	0.361	0.361	0.354	0.354	0.353	0.257	0.257	0.251	0.251	0.250	0.245	0.251
0	0.358	0.363	0.360	0.359	0.361	0.362	0.357	0.362	0.365	0.362	0.371	0.364	0.362	0.356	0.227	0.252	0.226	0.249	0.226	0.181	0.240
1	0.356	0.354	0.355	0.320	0.359	0.359	0.324	0.359	0.314	0.302	0.314	0.358	0.356	0.286	0.220	0.201	0.210	0.185	0.207	0.165	0.181
2	0.358	0.344	0.351	0.300	0.364	0.365	0.284	0.358	0.273	0.266	0.289	0.360	0.358	0.237	0.216	0.185	0.204	0.147	0.200	0.161	0.139
3	0.358	0.336	0.355	0.260	0.367	0.367	0.248	0.360	0.244	0.238	0.254	0.358	0.354	0.184	0.210	0.167	0.191	0.125	0.180	0.151	0.120
4	0.360	0.330	0.355	0.235	0.365	0.367	0.215	0.362	0.218	0.218	0.234	0.358	0.352	0.123	0.209	0.149	0.177	0.105	0.170	0.140	0.094
5	0.360	0.318	0.339	0.220	0.368	0.371	0.201	0.362	0.196	0.198	0.210	0.359	0.353	0.094	0.209	0.135	0.171	0.089	0.162	0.133	0.082
6	0.359	0.306	0.327	0.200	0.367	0.372	0.184	0.362	0.171	0.179	0.197	0.358	0.345	0.069	0.207	0.120	0.159	0.079	0.150	0.122	0.073
7	0.359	0.289	0.315	0.185	0.367	0.370	0.172	0.362	0.156	0.162	0.184	0.352	0.340	0.055	0.204	0.107	0.144	0.069	0.141	0.114	0.058
8	0.363	0.279	0.298	0.170	0.366	0.368	0.157	0.366	0.144	0.151	0.165	0.347	0.337	0.041	0.200	0.097	0.134	0.063	0.130	0.105	0.050
12	0.358	0.255	0.279	0.120	0.356	0.361	0.114	0.359	0.098	0.098	0.117	0.318	0.313	0.014	0.198	0.080	0.101	0.045	0.107	0.077	0.037
16	0.357	0.227	0.249	0.100	0.355	0.358	0.091	0.358	0.065	0.065	0.083	0.300	0.296	0.004	0.198	0.071	0.076	0.035	0.085	0.057	0.027
24	0.354	0.180	0.195	0.073	0.352	0.348	0.045	0.354	0.030	0.029	0.031	0.257	0.261	0.002	0.195	0.057	0.057	0.020	0.062	0.050	0.012
Time (h)	Absorbance of membranes under other lights																				
	Visible light			Solar-like light																	
	T-Co	TB2-Co	TC-Co	T-Co	TB2-Co	TC-Co															
00	0.354	0.354	0.35	0.33	0.331	0.331															
0	0.369	0.369	0.37	0.34	0.334	0.339															
2	0.361	0.354	0.35	0.29	0.316	0.306															
4	0.354	0.35	0.33	0.27	0.3	0.289															
6	0.349	0.346	0.33	0.25	0.294	0.271															
8	0.346	0.342	0.32	0.23	0.287	0.266															
10	0.344	0.339	0.32	0.22	0.278	0.262															

N, neat; T, TiO_2_; B2, 2 wt.% BiVO_4_; B20, 20 wt.% BiVO_4_; C, 2wt.% CNT; -PVP, 1 wt.% polyvinylpyrrolidone; -Bl, blended; -Co, coated; -Gr, grafted
